# Hollow Microcapsules as Periocular Drug Depot for Sustained Release of Anti-VEGF Protein

**DOI:** 10.3390/pharmaceutics11070330

**Published:** 2019-07-11

**Authors:** Krishna Radhakrishnan, Anita Vincent, Rini Rachel Joseph, Miguel Moreno, Andreas Dickescheid, Rupesh Agrawal, Subbu Venkatraman

**Affiliations:** 1 NTU-Northwestern Institute for Nanomedicine (NNIN), School of Materials Science and Engineering, Nanyang Technological University (NTU), 50, Nanyang Avenue, Singapore 639798, Singapore; 2 Tan Tock Seng Hospital, Novena, Singapore 308433, Singapore

**Keywords:** protein delivery, controlled release, anti-VEGF, microparticles, drug delivery, layer by layer microcapsules, age related macular degeneration, back of the eye

## Abstract

Diseases affecting the posterior segment of the eye such as age-related macular degeneration and diabetic retinopathy are leading causes of blindness all over the world. The current treatment regimen for such diseases involves repeated intravitreal injections of anti- Vascular Endothelial Growth Factor (VEGF) proteins. This method is highly invasive and can lead to severe complications. In an attempt to develop less invasive alternatives, we propose the use of a controlled release system consisting of anti-VEGF loaded hollow microcapsules that can be administered periocularly to form drug eluting depots on the episcleral surface. The microcapsules with either positive or negative surface charge were prepared by a layer by layer approach and showed pH responsive permeability switching. An ex vivo experiment using porcine sclera indicated positively charged microcapsules remained on the episcleral surface over four days while the negatively charged microcapsules were washed away. These positively charged microcapsules were then loaded with anti-VEGF protein ranibizumab using pH dependent permeability switching and protein release from the microcapsules were studied using an in vitro setup. An ex vivo experiment utilizing porcine sclera demonstrated sustained release of ranibizumab over seven days with zero-order kinetics.

## 1. Introduction

Diseases affecting the posterior segment of the eye such as age-related macular degeneration (AMD) and diabetic retinopathy (DR) are leading causes of blindness all over the world. Management of these diseases is often very challenging, primarily due to the various static and dynamic barriers associated with drug delivery to the posterior segment of the eye [1,2]. The current gold standard for treatment is intravitreal injection of anti-Vascular Endothelial Growth Factor (anti-VEGF) proteins and peptides, which directly delivers the drug into the vitreous humor. This technique overcomes various ocular barriers by directly delivering the drug at the site of action [3]. However, the invasive nature of the procedure has several drawbacks. Treating AMD and DR requires frequent intravitreal injections which has been shown to increase the possibility for complications such as endophthalmitis, cataract, retinal tears and retinal detachment [4,5]. Moreover, the pain and discomfort associated with intravitreal injections leads to poor patient compliance. The efficiency of treatment is further reduced by non-uniform diffusion kinetics and distribution profiles [6] mainly due to the suboptimal diffusion of high molecular weight anti-VEGF antibodies in the highly viscous vitreous humor. Although there have been attempts to introduce sustained release drug carriers for improved drug residence and prolonged therapeutic effect, almost all of them have been designed to be administered by intravitreal injections [4,7,8,9,10].

A convenient alternative to intravitreal injection is the non-invasive periocular route which involves introducing the drug to the subconjunctival, retrobulbar, posterior juxtascleral or subtenon spaces. Drugs administered into these spaces move to target tissue in the posterior segment of the eye via trans-scleral diffusion, systemic circulation or through anterior pathways [11,12,13], with trans-scleral diffusion being the most significant pathway [14]. While less invasive, the periocular route too suffers from limitations caused mainly due to the various static and dynamic barriers of the eye which hinders drug diffusion, enhances drug loss and reduces the efficacy of the treatment [15]. Moreover, the properties of the drug such as charge, hydrophilicity and size also plays an important role in their effective transport across the sclera [16,17,18,19,20].

In order to utilize the less invasive nature of periocular drug administration and to overcome the limitations of high drug loss due to dynamic barriers, we have evolved a strategy that involves encapsulating anti-VEGF proteins within polymeric carriers that can then be injected periocularly to form a drug eluting depot on the episcleral surface. In the present study, we explore the feasibility of encapsulating anti-VEGF proteins within hollow polymeric carriers intended for subconjunctival injections. The carrier design and the fabrication processes are chosen carefully such that (i) the carrier can be retained on the sclera surface for extended time periods, (ii) protein stability is maintained during fabrication and administration, and (iii) the functional drug is released slowly from the carrier.

**Figure 1:**
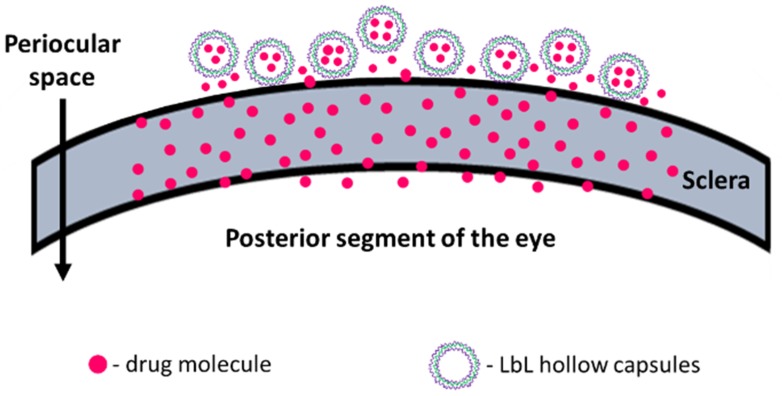
Schematic representation of periocular drug depot formed by layer by layer (LbL) hollow capsules.

The sclera is made up of a network of collagenous tissue, embedded with negatively charged proteoglycans and a small number of scattered cells. The sclera thus acts as a negatively charged porous membrane with numerous water channels. In order to form a drug depot, the particle should then either penetrate into the sclera to form an intrascleral drug depot or stick to the episcleral surface to form a periocular depot (Figure 1). To achieve this, we designed our carriers to have a positively charged surface, thus facilitating attractive interactions with the negatively charged sclera. Carrier size was also controlled to obtain 0.2 to 10 µm diameter particles as studies show that the preferred size for periocular retention of particles is between 0.2 µm and 10 µm [16,21]; particles below this size range enter the circulation and are removed quickly from the site of injection while particles above it can cause irritation in the eye after injection. The carrier was fabricated using an aqueous based layer by layer assembly (LbL) approach carried out under very mild conditions so as to preserve the functional stability of the protein. The protein release from the carriers were then evaluated in an in vitro set up as well as ex vivo sclera set up.

The polymeric carrier was fabricated using two clinically used biopolymers: protamine sulfate (PRM) and carboxymethyl cellulose (CMC). PRM is an FDA approved drug used to reverse heparin toxicity. Due to its cationic nature and inherent biocompatibility, it has been widely explored as a cellular uptake enhancer and transfection vector. PRM is made of predominantly positively charged amino acids specifically arginine which contributes to more than 60% of the total amino acids of PRM. This provides it with a net positive charge at physiological pH values with its isoelectric point (pI) estimated to be above 10 [22].

CMC is a modified form of cellulose in which the hydroxyl groups of the glucopyranose monomers of the cellulose backbone are attached to carboxymethyl groups. The carboxymethyl groups provide the polymer a net negative charge with a pKa of around 4.3. CMC is widely used as an emulsifying agent and thickener in pharmaceutical formulations. Hence this combination is expected to be biocompatible. In this study we utilize PRM and CMC to synthesize layer by layer assembled hollow capsules and evaluate their ability to act as anti-VEGF drug depots for treatment of AMD using the periocular route.

## 2. Materials and Methods 

Protamine sulfate salt from salmon (PRM, *M*_w_ ~5.1 kDa), carboxymethyl cellulose (CMC, *M*_w_ ~250 kDa), poly (styrene sulfonate) (PSS, *M*_w_ = 70 kDa), sodium carbonate, calcium chloride, ethylene diamine tetra-acetic acid (EDTA), rhodamine, rhodamine-BSA, and phosphate buffer saline (PBS) were bought from Sigma Aldrich, Singapore. Porcine scleral tissue was obtained from a local abattoir (Primary Industries, 2 Buroh Lane, Singapore) with permission from the Agri-food and Veterinary Association (AVA), Singapore (BPN Approval number: BPN-0015-2013-MSE, 7 March 2013). Optimal cutting temperature (OCT) compound was bought from Leica microsystems (Teban garden crescent, Singapore) and Vectorshield^®^ mounting medium with DAPI was bought from Vector Laboratories (San Diego, CA, USA).

### 2.1. Preparation of CaCO_3_ Microparticles

PSS doped CaCO_3_ microparticles were prepared to be used as templates to form the spherical hollow microcapsules. 2 mg/mL PSS was dissolved in 0.33 M CaCl_2_ and the solution was stirred using a magnetic stirrer for 10 min. To this, an equal volume of 0.33 M Na_2_CO_3_ was added rapidly to produce a white precipitate containing CaCO_3_ microparticles. This reaction was allowed to continue at room temperature for another 5 min. The resulting CaCO_3_ microparticles (white precipitate) was washed and collected using a vacuum filtration apparatus with cellulose acetate membrane of 0.4 µm pore size. The microparticles were collected on the surface of the membrane while the unreacted reactants passed through the filter. These particles were then washed by using deionized (DI) water and dried at 50 °C and stored until further use.

### 2.2. Fabrication of Layer by Layer Assembled Hollow PRM/CMC Microcapsules

The hollow microcapsules were fabricated by layer by layer assembly which involves alternate deposition of oppositely charged polyelectrolytes on to a particle which acts as the template. In the present study, we have fabricated PRM/CMC hollow microcapsules using CaCO_3_ microparticles as templates. 10 mg/mL of CaCO_3_ microparticles were washed by centrifugation and resuspended in DI water. The washed particles were then incubated with 1 mg/mL solution of PRM for 15 min. This was followed by washing and centrifugation to remove unabsorbed PRM and the particles were then incubated in 1 mg/mL CMC solution. This was again washed and centrifuged to get CaCO_3_ particles coated with PRM/CMC layers. The incubation processes were repeated till the desired number of layers were obtained. The LbL coated particles were subsequently treated with 0.2 M EDTA to remove the CaCO_3_ template to obtain hollow PRM/CMC microcapsules.

### 2.3. Characterization of Microcapsules

The size and zeta potential of the hollow PRM/CMC microcapsules were measured using Malvern Zetasizer ZS™ (Malvern Instruments, Worcestershire, UK).

### 2.4. Scanning Electron Microscopy

Scanning electron microscopy (SEM) was used to study morphology of the microcapsules. PRM/CMC microcapsules suspended in water were placed on a silica wafer and air dried before being coated with gold to improve their conductivity. The air-dried gold coated samples were then visualized using scanning electron microscopy fitted with a field emission gun (JSM-6340FESEM, JEOL Ltd, Tokyo, Japan). The images were obtained at 7 kV with a working distance of 10 cm.

### 2.5. pH Dependent Permeability Characterization

In order to profile the pH responsive properties of the PRM/CMC microcapsules, a sample containing the capsules was placed in an Eppendorf tube. The pH of this sample was adjusted, and the sample was then co-incubated with either Rhodamine or Rhodamine-BSA. The location of the dyes on the sample was visualized using confocal laser scanning microscopy (CLSM, Zeiss LSM 510 Meta, Jena, Germany).

### 2.6. Anti-VEGF Protein Loading into PRM/CMC Microcapsules

Anti-VEGF protein ranibizumab was loaded into hollow PRM/CMC microcapsules by incubating the capsules in protein solutions of different concentrations (1 and 0.5 mg/mL). Prior to incubation, pH of the capsules was adjusted to 5 to make them permeable and the procedure was carried out at room temperature for different time periods. After the required time, the samples were removed from the protein solution and their pH was again adjusted to pH 7 to lock the capsules. The samples were then centrifuged to separate the unloaded free protein from the protein encapsulated capsules for further analysis.

The encapsulation efficiency was calculated by the following equation:

$$\mathrm{Encapsulation}\;\mathrm{Efficiency}\;\left(\%\mathrm{EE}\;\right)=\;\frac{{\mathrm{Drug}}_{\mathrm{total}}{-\;\mathrm{Drug}}_{\mathrm{supernatant}}}{{\mathrm{Drug}}_{\mathrm{total}}}\;\times \;100\%$$

The loading capacity was calculated using the following equation:

$$\mathrm{Loading}\;\mathrm{Capacity}\;\left(\%\mathrm{LC}\;\right)=\;\frac{{\mathrm{Drug}}_{\mathrm{total}}{\;-\;\mathrm{Drug}}_{\mathrm{supernatant}}}{\mathrm{weight}\;\mathrm{of}\;\mathrm{drug}\;\mathrm{loaded}\;\mathrm{particles}}\;\times \;100\%$$

where Drug_total_ represents the total amount of protein (in mg) added initially to the PRM/CMC hollow capsule solution and Drug_supernatant_ represents the amount of un-encapsulated free drug in the supernatant.

### 2.7. FITC Labelling of Ranibizumab and Localization Studies

Drug loading and localization of the drug within the PRM/CMC microcapsules was studied by using CLSM. To visualize the ranibizumab localization under CLSM, the protein molecule was tagged with fluorescein isothiocyanate (FITC) as a fluorescent probe. 2 mg/mL protein was dissolved in sodium bicarbonate buffer at pH 9 and purified by running through a Sephadex PD desalting column to remove potential contaminants. To 1 mL of this purified protein sample, 50 µL of 1 mg/mL FITC dissolved in DMSO (freshly prepared) was added slowly in aliquots of 5 µL under gentle stirring. The resulting solution was incubated for 8 h at 4 °C in the dark. The reaction was then stopped using NH4Cl and the resulting protein–dye conjugates were separated from unbound dye by running them through Sephadex PD gel matrix. The purified bound protein was quantified absorbance spectroscopy and stored at 4 °C until further use.

The FITC labelled ranibizumab was incubated in a solution containing PRM/CMC microcapsules at pH 4.5 for 12 h. The un-encapsulated FITC labelled Ranibizumab was separated from the drug loaded particles by centrifugation and washing steps.

### 2.8. Release Experiments Setup

In vitro release assays were carried out in dialysis membranes with a molecular weight cutoff of 1000kDa. Drug loaded microcapsules placed within the dialysis membranes were then placed inside glass bottled containing 10 mL of the release media (PBS at pH 7.4) mimicking physiological conditions. 0.05% (w/v) sodium azide was added to the release media to avoid contamination. The release media was then collected at various time points and replaced with fresh release media. The concentration of proteins in the release media was calculated by fluorescence spectroscopy using a Varian Cary Eclipse spectrophotometer (Agilent Technologies, Victoria, Australia). The excitation wavelength was set at 280 nm and the emission was recorded at 340 nm. The concentration of the analyte solution was calculated by extrapolating from a standard curve made with varying concentrations of ranibizumab.

### 2.9. Ex Vivo Porcine Sclera Tissue Isolation

Scleral tissue from freshly excised porcine eyes (Primary Industries, 2 Buroh Lane, Singapore) was used to study the trans-scleral transport of rhodamine loaded PRM/CMC hollow capsules. Permission and approval from the Institutional Biosafety Committee at the Nanyang Technological University was obtained for all experiments involving sclera.

After external muscle tissue was removed, a circumferential cut was made along the limbus to remove the anterior segment of the eye and the vitreous humor. The retina was removed as well and the remaining layers of tissue was cut into two halves along the center of the optic nerve. The choroid layer was then gently scrapped off and the sclera was rinsed with PBS (Sigma Aldrich, 0.01 M phosphate buffer, 0.0027 M potassium chloride and 0.137 M sodium chloride, pH 7.4, at 25 °C) containing 0.05% sodium azide. The tissue was then trimmed into circular pieces (~1cm diameter) such that the thickness of the sections ranged from 0.8 to 1.2 mm.

### 2.10. Sclera Transport Study Setup

The circular sections of sclera isolated from the porcine eyes were placed within tissue holders and mounted in Ussing chambers (Navicyte, Warner Instruments, Hamden, CT, USA) such that the vitreous side of the sclera was in contact with 2 mL of PBS containing 0.05% sodium azide in the receiver chamber. Around 400 uL of the rhodamine loaded LbL under study was then placed on the episcleral side of the sclera and the setup was left undisturbed for 4 days. The Ussing chambers were maintained at 37 °C using a circulating water bath connected to a heater block.

At the end of 4 days, the scleral sections were removed from the Ussing chambers and submerged in 4% paraformaldehyde (PFA) overnight. The fixed tissue was then rinsed with PBS before embedding in optimal cutting temperature (OCT) compound. Sections with thickness of 5 µm were obtained from the embedded tissue using a microtome-cryostat and stained with DAPI. The sections were observed under an epi-fluorescent microscope to locate the LbL particles in the sclera after 4 days as a study of their transport.

Scleral transport of bare and encapsulated ranibizumab was also carried out using the trans-scleral transport setup. The samples under study were placed on the episcleral surface and the amount of protein transported was measured over a 7 day time period using enzyme-linked immunosorbent assay (ELISA). The buffer in the receiver chamber was replaced at each time point to ensure sink conditions. Permeability coefficient (cm/s) of both bare and encapsulated ranibizumab was calculated using the following equation [23]:

$$\mathrm{Permeability}\;\mathrm{coefficient}=\;\frac{Q}{A\;\times \;C_{i}}$$

where Q is the steady state transport rate of protein across the sclera as obtained from the linear slope of cumulative trans-scleral ranibizumab transport, A is the surface area of sclera and C_i_ is the initial concentration of protein placed in the episcleral surface.

### 2.11. Enzyme-Linked Immunosorbent Assay (ELISA) for Measuring Functional Protein Release

ELISA plates were prepared by coating VEGF onto polystyrene well-plates. The VEGF coated polystyrene plates (Thermo Scientific) were prepared by incubating the wells overnight with 10 µg of VEGF dissolved in 100 µL of 50 mM carbonate buffer (pH 9.6) at 4 °C. After incubation, the plates were washed 3 times with 0.05% Tween-20 dissolved in PBS. Subsequently, the wells were blocked with 2% (w/v) BSA in PBS with 0.05% (v/v) Tween-20 pH 7.4 overnight at 4 °C. The plates were then washed thrice with 0.05% Tween-20 dissolved in PBS and dried prior to use for protein quantification. In order to analyze the functional protein released, samples were added to the respective well plates and incubated for 2 hat room temperature. The wells were washed five times with 0.05% (v/v) Tween-20 in PBS to remove any unabsorbed proteins from the wells. A secondary antibody (Goat anti-human IgG, 1:20,000 dilution) was subsequently added to the wells and incubated for 45 min at room temperature. The plates were then washed five times with 0.05% (v/v) Tween-20 in PBS and 100 µL of Supersignal^®^ ELISA Pico Chemiluminescent substrate buffer (Thermo Scientific) were added per well. The ensuing luminescence was measured immediately using a microplate reader (TECAN microplate reader infinite200, Salzburg, Austria). The detection limit of this assay was approximately 0.75 ng/mL.

## 3. Results

**Figure 2:**
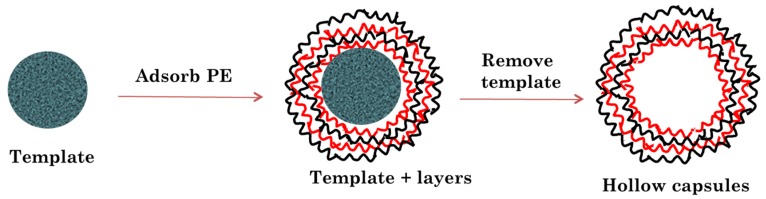
Schematic representation of hollow microcapsule fabrication using template assisted layer by layer assembly process.

In the present study, we have developed layer by layer assembled hollow capsules as a carrier for controlled release of anti-VEGF proteins for treatment of AMD. The layer by layer assembly involves adsorption of oppositely charged polymers on to specific templates which are later etched away to obtain the hollow capsule structure (Figure 2). As templates, we utilized CaCO_3_ microparticles with a diameter of 5 µm due to the mild and bio-friendly conditions that can be used for their removal [24]. The polyelectrolytes were chosen based on their biocompatibility and charge at different pH values. The polymers should ideally display appropriate charges at the encapsulation pH which in turn should match the pH range at which the protein is stable. 

Moreover, the polyelectrolytes should attain suitable charge at physiological pH to facilitate protein release. Based on this, PRM and CMC were chosen which had a pI of >10 and pka of 4.3 respectively.

**Figure 3:**
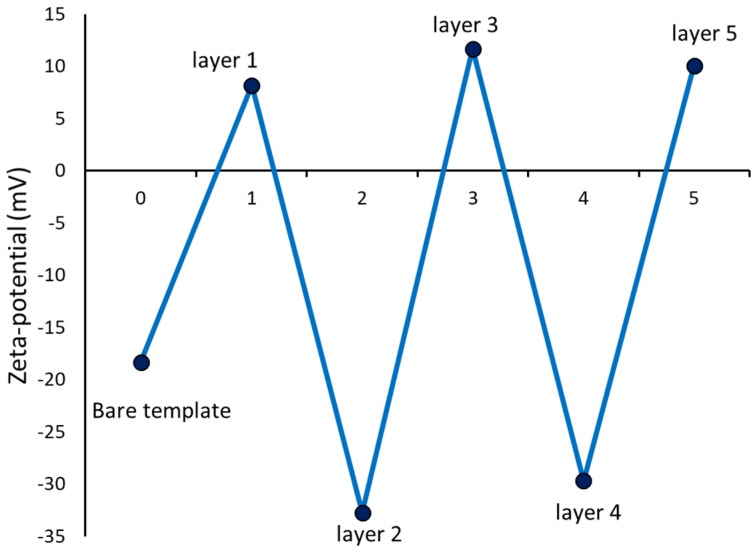
Alternation of zeta potential of the CaCO_3_ template upon adsorption of various polyelectrolyte layers. The odd layers are made up of protamine sulfate (PRM) and the even layers consist of carboxymethyl cellulose (CMC).

The gold standard for following the successful completion of the various steps in LbL assembly is to follow the zeta potential changes at each step of polyelectrolyte adsorption [25]. The zeta potential of the bare CaCO_3_ template was observed to be −18 mV and hence the first layer to adsorb was the positively charged PRM. Subsequently, CMC was adsorbed and the LbL process was repeated till the required layers of PRM and CMC was coated on the CaCO_3_ microparticles. The progress of the LbL assembly of PRM and CMC on the CaCO_3_ template can be observed from the variation observed in the zeta potential after each adsorption step (Figure 3).

Two different sets of hollow capsules were prepared with different outer charges to evaluate the effect of outer charge on the interaction and transport through the scleral tissue. In order to obtain a net negative surface charge, two layers of PRM and two layers of CMC were coated alternatingly which resulted in the negatively charged CMC forming the outer layer. The positively charged hollow capsules were fabricated by coating three layers of PRM and two layers of CMC alternatingly on CaCO_3_ microparticles.

Upon successfully coating the required number of polyelectrolyte layers, the temporary core was etched using a mild and aqueous etching procedure involving EDTA dissolved in pH 7 water. EDTA is a chelating agent that complexes with the Ca ions present in the CaCO_3_. The Ca–EDTA complexes are water soluble and hence can be separated from the hollow polymeric capsules by centrifugation steps.

**Figure 4:**
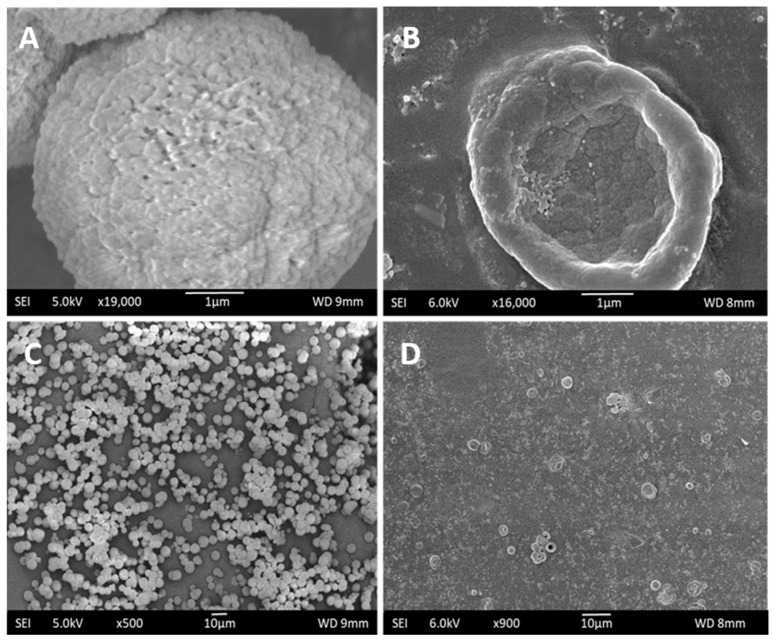
SEM images of CaCO_3_ microparticles that were fabricated for use as templates for LbL assembly (**A**,**C**) and hollow PRM/CMC microcapsules after core removal (**B**,**D**).

Figure 4 shows the SEM images of air dried CaCO_3_ particles before the LbL assembly and the final hollow capsules after CaCO_3_ template removal. The hollow capsules assumed a collapsed and flattened morphology upon drying indicating the removal of the solid core. The hollow capsules had a hydrodynamic size of around 3000 nm.

### 3.1. Transport Studies

**Figure 5:**
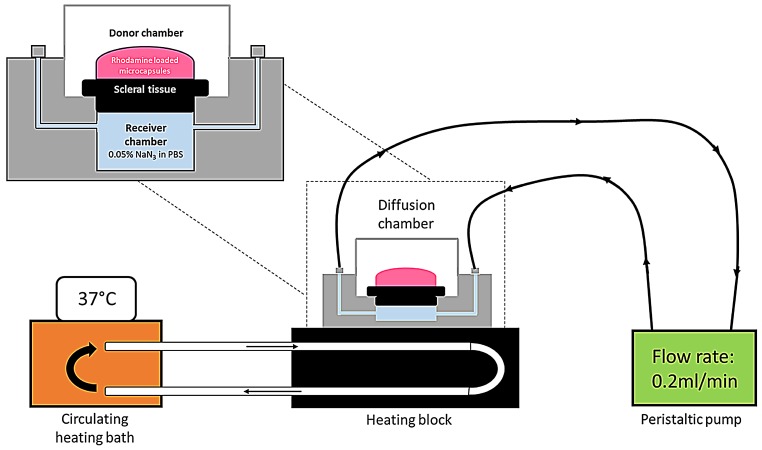
Schematic representation of trans-scleral transport experiment; (**A**) setup of sclera and sample in horizontal Ussing chamber.

**Figure 6:**
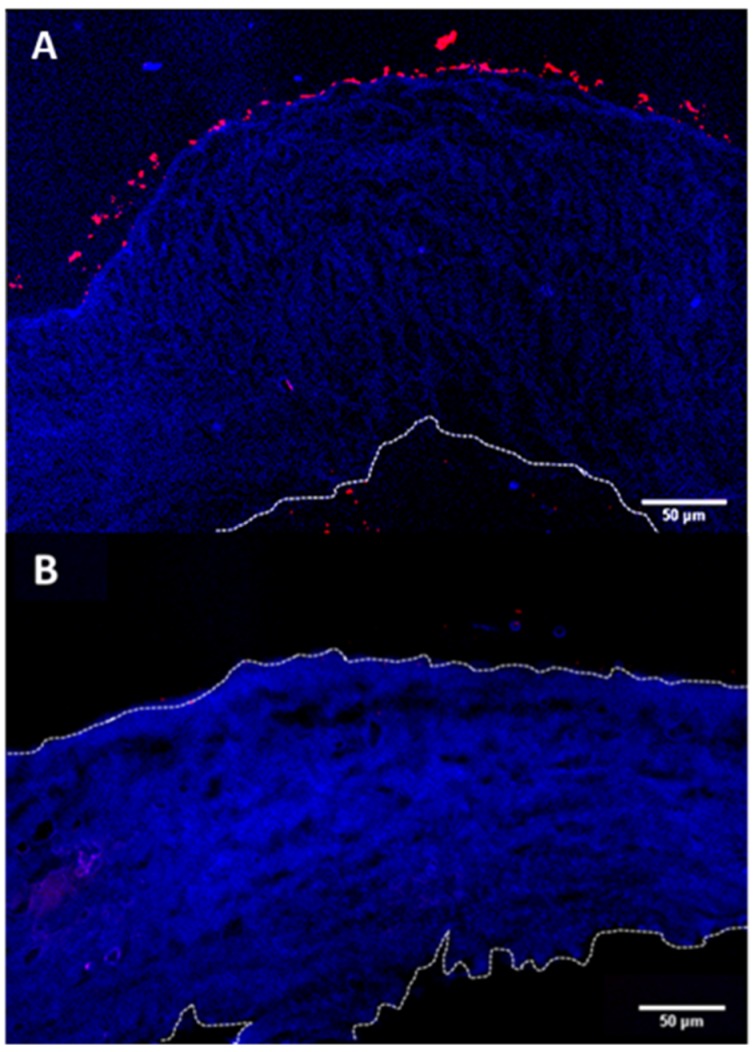
Trans-scleral transport images showing (**A**) positive LbL particles sticking to episcleral surface and (**B**) no negative particles on episcleral surface.

The prime objective of the present study is to fabricate hollow capsules that can be used as drug eluting depots for controlled release of anti-VEGF proteins. The key requirement for such a system is that the depot must stick either to the surface of the sclera or stay immobilized within the sclera itself. This is expected to help prevent the clearance of the carriers from the periocular space. In order to study the interaction of the hollow PRM/CMC capsules with the sclera, an ex vivo porcine sclera model was used. The excised porcine sclera was mounted on to a horizontal Ussing chamber (Figure 5). The fluorescently labelled PRM/CMC microcapsules (red fluorescence from rhodamine) were loaded into the donor chamber of the transport setup in direct contact with the episcleral surface of the sclera tissue. This was to mimic the in vivo condition after subconjunctival injection where the particle is directly deposited onto the episcleral surface. At the end of the experiment, the tissue samples were taken from the transport set up, cryo-sectioned and imaged using a fluorescent microscope. From the fluorescent images taken after four days, the positively charged PRM/CMC capsules were seen sticking to the episcleral surface while the negatively charged capsules were completely absent (Figure 6). Neither type of hollow capsules were able to transport into the sclera even after four days of incubation. This could be explained in terms of the “sieving action” of the sclera whereby only neutral, nanosized particles penetrate through the negatively charged collagen fibrils present within the sclera while particles bigger than 1 micron remain outside.

However, despite the absence of trans-scleral transport, the positively charged hollow capsules were found to adhere to the episcleral surface due to electro-attractive interactions with the negatively charged episcleral surface while the negatively charged particles did not. In in-vivo conditions, this could translate into longer persistence of the positively charged hollow capsules in the periocular space, leading to the formation of a stable periocular drug depot in in-vivo conditions. Hence, we continued our further studies with the positively charged hollow capsules.

### 3.2. Hollow Capsule Wall Permeability Studies

Once the ability of positively charged hollow capsules to act as drug loaded depots by attaching to the episcleral space was demonstrated, the study focused on loading the capsules with the therapeutic anti-VEGF protein. We utilized the pH dependent permeability changes usually observed in LbL assembled hollow capsules to achieve optimal loading.

**Figure 7:**
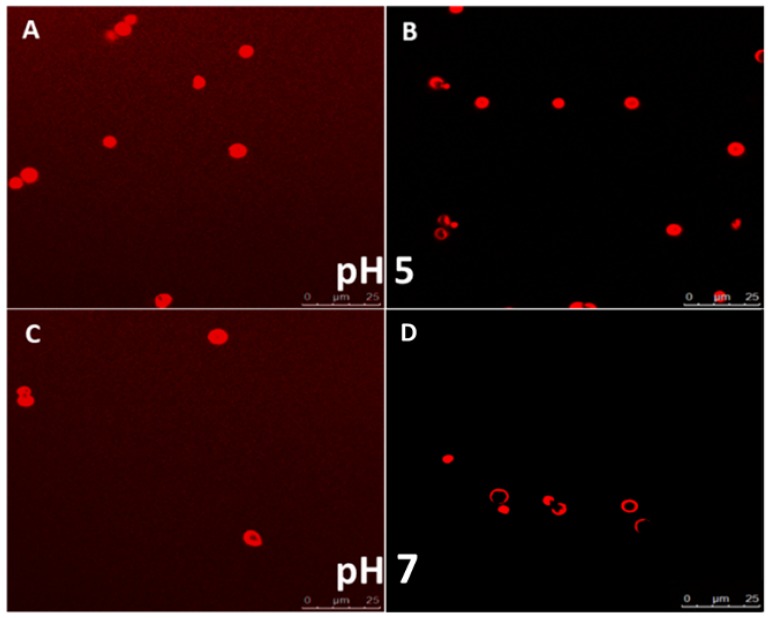
CLSM images showing pH dependent permeability changes at pH 5 for (**A**) rhodamine and (**B**) BSA-TRITC and at pH 7 for (**C**) rhodamine and (**D**) BSA-TRITC.

Due to the electrostatic nature of interaction between the polymer layers, LbL assembled polyelectrolyte capsules are known to be sensitive to environmental pH changes. The permeability of these particles can be modified by adjusting the local pH of the medium in which they are suspended. The permeability profile of the capsules depends on the pKa values of the respective polyelectrolytes used to build the layers. The profiling was done by incubating fluorescent molecules (a model protein, BSA) of different molecular weights with the capsules at different pH values. As observed in CLSM images (Figure 7A,C), the capsules were permeable to Rhodamine (*M*_w_ ~480 Da) and Rhodamine labelled BSA (*M*_w_ ~66.5 kDa) at pH 5. This is due to the fact that at pH 5, the carboxyl groups of CMC are mostly de-protonated. The de-protonation leads to diminished electrostatic interaction between the polyelectrolyte layers, allowing the walls of the microcapsules to be more permeable. This behavior was ideal for our application as the protein was known to be stable at this pH and hence these conditions can be used for loading the protein while preserving its functionality.

Interestingly, while the capsules were still permeable to rhodamine at pH 7, the rhodamine labelled BSA molecules were not able to reach the capsule core at pH 7 (Figure 7B,D). This indicates that apart from the pH sensitive variability in the permeability, the capsules show size selective permeability at various pH values. Since highest capsule permeability was observed at pH 5, we further utilized this pH for the loading experiments with the anti-VEGF protein Ranibizumab. Additionally, the data indicated that the capsules were suitable for retaining and slowly releasing larger molecules such as proteins at the physiologically relevant pH of 7.

### 3.3. Encapsulation of Anti-VEGF Agent Ranibizumab

Anti-VEGF proteins are antibodies or their fragments that can bind to and block VEGF, thereby inhibiting neovascularization. Ranibizumab which consist of the Fab portion of the anti-VEGF antibody bevacizumab (Avastin™) is the first anti-VEGF agent that was FDA approved for treatment of diabetic macular edema (DME). 

Unlike designing carriers for small molecular weight drugs, there are several critical considerations to be considered while designing carriers for therapeutic proteins. Of prime importance is the preservation of functional and structural stability of the protein during and after carrier fabrication and drug loading. This mandates that the particle fabrication and drug loading process must avoid strong chemical and physical agents that can destroy the functionality of the protein. In a previous study, we have evaluated the stability of Ranibizumab in the presence of various organic solvents and at different pH values. This extensive study, which consisted of a detailed evaluation of the structural and functional stability of the protein, provided a foundation for choosing the carrier system and deciding favorable conditions for drug loading [3]. Based on the results we have observed in this previous study and the results of the permeability studies, pH 5 was chosen for loading the protein into the positively charged PRM/CMC capsules.

**Figure 8:**
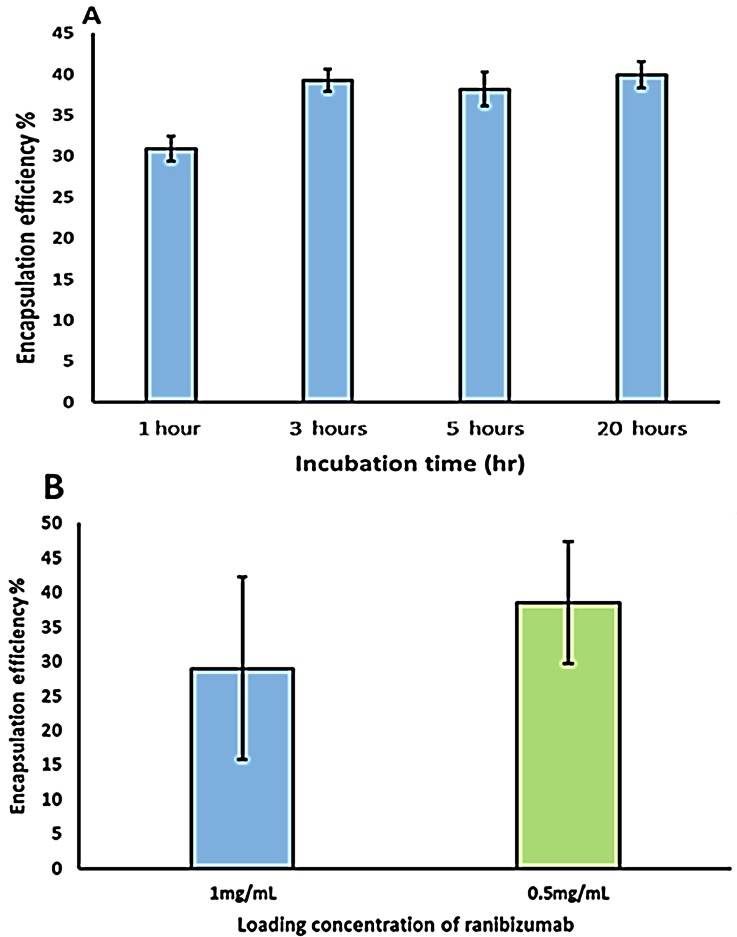
Effect of incubation time (**A**) and initial protein concentration (**B**) on ranibizumab encapsulation in capsules.

Ranibizumab loading was carried out by adding a known amount of ranibizumab to pH adjusted capsule solution. This solution was kept on a shaker for different time points and the final loaded capsules were separated from the un-encapsulated protein molecules by centrifugation. The results of the encapsulation studies (Figure 7) indicate that encapsulation efficiency of the Ranibizumab in the hollow capsules is dependent on the initial drug concentration. The encapsulation efficiency was observed to be 29% when the initial drug concentration was 1 mg/mL which increased to 38% when the initial ranibizumab concentration was reduced to 0.5 mg/mL. The encapsulation efficiency was also found to be dependent on the time of incubation (Figure 8), with encapsulation efficiency increasing from 30% with 1 h incubation to 38% with 3 h incubation. Interestingly, no further improvement in encapsulation efficiency was observed after incubation for increased time periods, indicating a spontaneous deposition mechanism rather than a slow diffusion.

**Figure 9:**
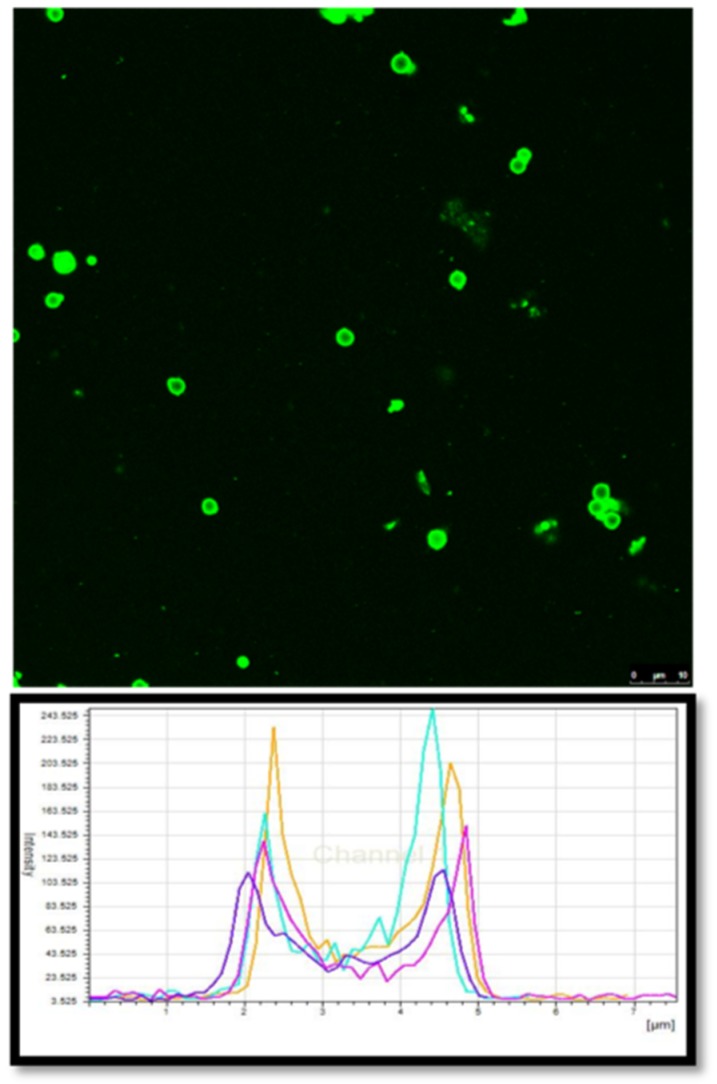
CLSM image of ranibizumab–fluorescein isothiocyanate (FITC) loaded PRM/CMC and their fluorescence intensity profile.

The driving force for this phenomenon can be ascribed to the charge interaction between ranibizumab and the oppositely charged polyelectrolytes that are present on the walls of the hollow capsules. In order to further corroborate this hypothesis and to understand the colocalization of the protein within the hollow capsules, we tagged ranibizumab with FITC and loaded the fluorescent labelled protein into the hollow capsules. The CLSM images (Figure 9) of the drug loaded PRM/CMC capsules showed that the green fluorescence emitted by the FITC tagged ranibizumab could be seen within the hollow capsules. Interestingly, a higher fluorescence intensity was observed on the hollow capsule walls compared to the core indicating preferential entrapment of the protein within the polyelectrolyte layers in comparison to the hollow core. Previous studies report that the LbL assembled hollow capsules have polyelectrolyte chains in their walls which are only partially charge compensated [26]. The uncompensated part of the chain is still available to trap the charged protein molecules by electrostatic interactions. This kind of interaction will be useful for both enhancing drug loading and also for sustaining the drug release over longer time periods.

### 3.4. In Vitro and Ex Vivo Release Studies with PRM/CMC Encapsulated Ranibizumab 

**Figure 10:**
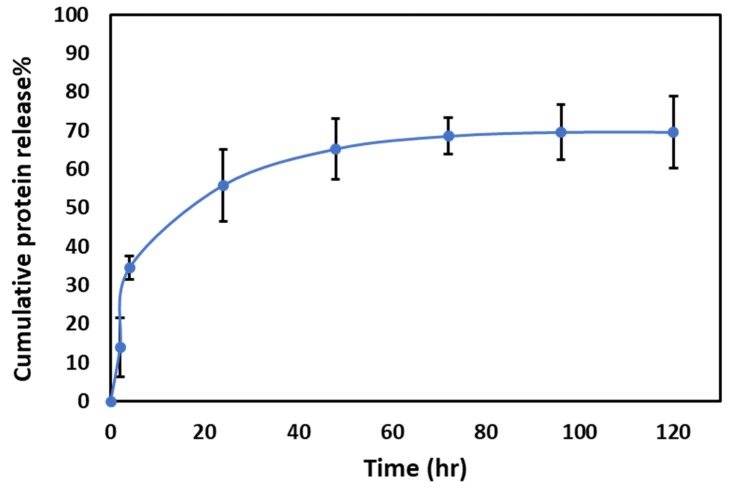
In vitro release profile of PRM/CMC hollow capsules in phosphate buffer saline (PBS) 7.4 incubated at 37 °C.

To better understand the protein release kinetics from the PRM/CMC capsules, we carried out in vitro release studies using PBS as a release media. The in vitro release profile (Figure 10) showed that the PRM/CMC hollow capsules are capable of facilitating sustained release of ranibizumab over five days, making it a viable system for periocular drug delivery.

**Figure 11:**
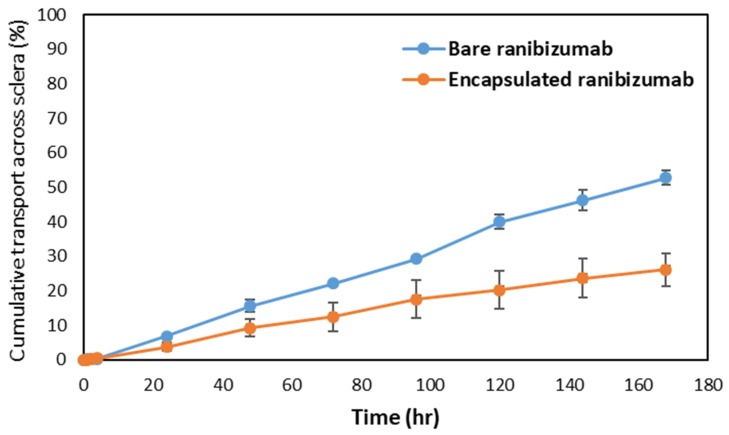
Release and transport of bare and encapsulated ranibizumab across sclera over seven days; similarity factor f2 = 44.10 indicating significant difference between release profiles.

To understand the release mechanism of ranibizumab loaded hollow capsule in-vivo, a comparative study of the release and transport of ranibizumab across the sclera was carried out. The study compared release and transport of encapsulated and bare ranibizumab using ELISA in order to understand the amount of functional ranibizumab reaching the target. At the end of the study, it was observed that while 52% of bare ranibizumab had crossed the sclera and reached the receptor chamber, only 26% of the encapsulated ranibizumab was released and transported (Figure 11). The ex vivo release showed a statistically significant difference between the encapsulated and the bare protein, wherein the encapsulation resulted in reducing the rate of release and transport by a factor of 2. Similarity factor f2 of the release profiles was found to be 44.10, indicating significant difference between bare and encapsulated ranibizumab [27]. The permeability coefficient of encapsulated ranibizumab was also found to be lower than bare protein by an order of 1000 which signified the ability of the hollow microcapsules to slowly release ranibizumab across sclera (Table 1).

**Figure 12:**
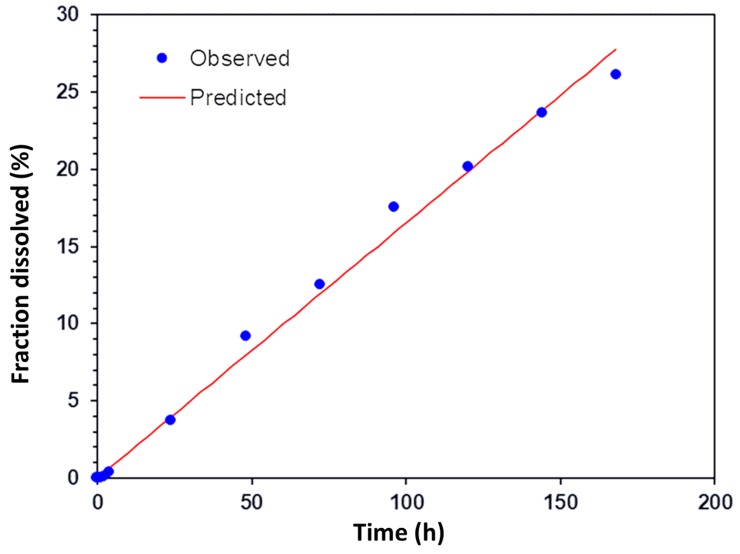
Fit of release and transport of encapsulated ranibizumab across sclera against zero-order release model; *R*^2^ = 0.9926.

The scleral transport profile of encapsulated ranibizumab was also fitted against different release kinetics models to further understand the transport mechanism. This was carried out using the DD solver Excel add in program [28]. The profile was found to fit the zero-order release model (Figure 12, *R*^2^ = 0.9926), indicating constant drug transport rate across the sclera from a depot. These results indicate that the PRM/CMC hollow capsules in conjunction with the sclera was able to facilitate sustained transport of ranibizumab across the sclera. The ex vivo transport studies were only carried out for seven days due to the limitations in the viability of the sclera tissue.

## 4. Discussion

The present study investigates an approach to substitute invasive and painful intravitreal injections by using a polymeric drug carrier that can be injected subconjunctivally to act as a drug eluting depot sitting on the episcleral surface. The study demonstrates the fabrication of hollow microcapsules loaded with anti-VEGF drug ranibizumab. The charge on the particle outer surface was shown to be a determining factor for successful adhesion of the particles to scleral surface. Positively charged hollow capsules adhered to the scleral surface while the negatively charged hollow capsules were absent near the sclera surface. We further demonstrated the successful loading of ranibizumab which was concentrated within the microcapsule walls. The in vitro release profiles of the microcapsules showed sustained release that lasted for five days. The ex vivo studies showed that the transport of ranibizumab is significantly slower when encapsulated compared to free ranibizumab. These results support our hypothesis that positively hollow microcapsules can act as drug eluting depots that can attach to the episcleral surface and slowly release anti-VEGF protein drugs. This kind of a drug delivery system has great promise for improving and replacing the current treatment methods, such as intravitreal injections, for posterior segment diseases. 

## Figures and Tables

**Table 1:** Permeability coefficient (cm/s) of bare and encapsulated ranibizumab.

Drug	Permeability Coefficient (cm/s)
Bare ranibizumab	5.09 × 10^−4^
Encapsulated ranibizumab	2.55 × 10^−7^
